# Comparison of bacterial profiles in human milk from mothers of term and preterm infants

**DOI:** 10.1186/s13006-023-00563-3

**Published:** 2023-06-08

**Authors:** Kumiko Miura, Miori Tanaka, Midori Date, Mizuho Ito, Noriko Mizuno, Katsumi Mizuno

**Affiliations:** 1 The Nippon Foundation Human Milk Bank, Nihonbashi-koamicho Square Building 1F, 17-10 Nihonbashi-koamicho, Chuo-ku, Tokyo, 103-0016 Japan; 2 Faculty of Medicine, Oita University, 1-1 Idaigaoka, Hasama-machi, Yufu-shi, Oita, 879-5593 Japan; 3 Japan Human Milk Bank Association, 4-4 Nihonbashi-Hisamatsucho, Chuo-ku, Tokyo, 103-8480 Japan; 4 Department of Pediatrics, Showa University of Medicine, 1-5-8 Hatanodai, Shinagawa-ku, Tokyo, 142-8666 Japan

**Keywords:** Neonatal intensive care unit, Human milk, Donor milk, Human milk microbiota, Bacteriological test

## Abstract

**Background:**

Reducing the disposal of donated human milk (HM) is important for efficient management of human milk banks (HMBs). The presence of bacteria growth is the main factor that contributes to the disposal of donated HM. The bacterial profile in HM is suspected to differ between term and preterm mothers, with HM from preterm mothers containing more bacteria. Thus, elucidation of the causes of bacterial growth in preterm and term HM may help to reduce the disposal of donated preterm HM. This study compared the bacterial profiles of HM between mothers of term infants and mothers of preterm infants.

**Methods:**

This pilot study was conducted in the first Japanese HMB, which was initiated in 2017. This study analyzed 214 human milk samples (term: 75, preterm: 139) donated by 47 registered donors (term: 31, preterm: 16) from January to November 2021. Bacterial culture results in term and preterm HM were retrospectively reviewed in May 2022. Differences in total bacterial count and bacterial species count per batch were analyzed using the Mann–Whitney U test. Bacterial loads were analyzed using the Chi-square test or Fisher’s exact test.

**Results:**

The disposal rate did not significantly differ between term and preterm groups (*p* = 0.77), but the total amount of disposal was greater in the preterm group (*p* < 0.01). Coagulase-negative Staphylococci, *Staphylococcus aureus*, and *Pseudomonas fluorescens* were frequently found in both types of HM. *Serratia liquefaciens* (*p* < 0.001) and two other bacteria were present in term HM; a total of five types of bacteria, including *Enterococcus faecalis* and *Enterobacter aerogenes* (*p* < 0.001) were present in preterm HM. The median (interquartile range) total bacterial counts were 3,930 (435–23,365) colony-forming units (CFU)/mL for term HM and 26,700 (4,050–334,650) CFU/mL for preterm HM (*p* < 0.001).

**Conclusions:**

This study revealed that HM from preterm mothers had a higher total bacterial count and different types of bacteria than HM from term mothers. Additionally, preterm infants can receive nosocomial-infection-causing bacteria in the NICU through their mother’s milk. Enhanced hygiene instructions for preterm mothers may reduce the disposal of valuable preterm human milk, along with the risk of HM pathogen transmission to infants in NICUs.

## Background

Human milk (HM) is recognized as the most ideal nutrient source for infants. In particular, preterm HM contains higher concentrations of nutrients such as proteins, fats, sodium, and other bioactive contents, compared with term human milk [1–3]. HM reduces the risks of necrotizing enterocolitis [4–6], sepsis, and other diseases; it also contributes to better long-term outcomes and infant neurodevelopment [4, 5]. In addition to nutrients and micronutrients, HM contains diverse types of bacteria and antibacterial agents. Various studies have investigated HM microbiota using culture methods and molecular approaches (e.g., metagenomic analysis). Bacteria in HM isolated by culture methods mainly include facultative anaerobes from the genera *Staphylococcus*, *Streptococcus*, *Enterococcus*, *Lactococcus*, *Leuconostoc*, *Weissella*, *Lactobacillus*, *Cutibacterium* (formerly *Propionibacterium*), and *Enterobacteriaceae* [6]. Metagenomic analysis using next-generation sequencing has recently enabled the detection of non-proliferative or non-viable bacterial cells, which could not be readily identified via culture methods; the results have helped to clarify the diversity and complexity of HM microbiota [7–10]. Metagenomic analysis has revealed bacteria that mainly belong to four phyla: *Firmicutes* (56.4%), *Proteobacteria* (17.3%), *Bacteroidetes* (14.7%), and *Actinobacteria* (11.6%). It has also identified bacteria such as *Lactobacillus salivarius*, *Lactobacillus fermentum*, *Bacteroides, Blautia*, *Clostridium*, *Collinsella*, *Coprococcus*, *Eubacterium*, *Acinetobacter*, *Bradyrhizobium*, and *Pseudomonas* [7–9].

There are several hypotheses for the origin of HM microbiota. Shirin et al. slightly modified the hypothesis of Fernández et al. [8] and proposed the presence of maternal microbiota. This paradigm involves the oro/enteromammary pathway (including translocation of both maternal oral bacteria and maternal gut bacteria) and the breast microbiota and exogenous microbiota; it also involves retrograde translocation (including maternal skin bacteria, infant oral bacteria, and breast pump-associated bacteria) and contamination related to human milk handling [9]. Although HM microbiota provides many benefits to infant health and development through multiple mechanisms [6, 9, 10], the presence of pathogenic microorganisms in HM carries a risk of infection, particularly for preterm infants.

There are several potential sources of pathogenic microorganism entry into HM, including expression of breast milk, storage of HM at home, shipping, processing in human milk banks (HMBs), and handling in neonatal intensive care units (NICUs). Among these sources, expression and storage at home are particularly influenced by the donor’s living environment conditions and hygiene [11].

Preterm HM is known to be better matched nutritionally for preterm infants. The presence of bacterial growth is the main reason for the disposal of donated HM [12]. To support more effective use of donated preterm HM, a better understanding of the bacterial profile in preterm and term HM is needed.

Based on the hypothesis that the type of bacteria in HM is related to the donor’s living environment, this study focused on differences in bacteria within HM between term donors whose infants were at home and preterm donors whose infants were hospitalized in NICU. The research aim was to compare the bacterial profiles of HM between mothers of term infants and mothers of preterm infants.

## Methods

### Design

This was a retrospective analysis of data from the Japan Human Milk Bank Association (JHMBA) from January to November 2021. The study compared bacterial culture results for HM between term maternal donors (37 to 41 weeks) and preterm maternal donors (< 37 weeks). This study was approved by the Showa University Research Ethics Review Board (Permit Number: 2714).

### Setting and participants

Forty-seven donors who registered at the JHMBA from January to November 2021 were included in the study. The donors’ health conditions were confirmed based on a health checklist. The donors were screened based on their detailed medical history, physical examinations, and laboratory data, in accordance with the Guidelines for the Establishment and Operation of a Donor Human Milk Bank 2018 [13]. Donors who did not meet the eligibility criteria were excluded from donating milk.

In Japan, term donors register themselves via HMB websites, whereas preterm donors are generally registered via referral from medical staff in NICUs.

The milk expression method was either by hand or with an electric or manual pump; expression equipment was provided free of charge by the JHMBA upon donor request. At the donor interview, the interviewer explained that after washing their hands, donors would wipe their breasts with cotton immersed in tap water, then express their breast milk in a clean environment. Expressed breast milk was collected in sterile soft plastic bags provided by the JHMBA and stored in a home freezer, separate from other food products. However, as explained above, detailed information regarding milk expression kits and processing procedures was not recorded. Milk was transported to the JHMBA in 1-L quantities within 1 month after expression, in conditions of –15 °C (or colder), through a shipping company contracted by the JHMBA. Upon arrival, all milk was checked to ensure that it remained frozen; any damage to sterile bags or presence of foreign matter was identified. Subsequently, the milk was stored at –20 °C for ≤ 3 months from the date of expression. The entire process followed the Guidelines for the Establishment and Operation of a Donor Human Milk Bank 2018 [13], which were compiled in accordance with Human Milk Banking Association of North America (HMBANA) guidelines [11]. All donated milk was pasteurized using the Holder pasteurization method (62.5 °C for 30 min); pre- and post-pasteurization samples were subjected to bacterial culture assessment.

The acceptance criteria for donated milk were total bacterial count of ≤ 10^5^ CFU/mL, *Enterobacteriaceae* count of ≤ 10^4^ CFU/mL, *Staphylococcus aureus* count of ≤ 10^4^ CFU/mL, and the absence of spore-forming bacteria. Donated HM was accepted as pasteurized donor HM if no bacteria were detected after Holder pasteurization. In addition to the strict safety standards, hygienic instructions for adequate expression, freezing, and shipping were provided to all donors by JHMBA staff at the time of registration.

### Measurements

Frozen milk was thawed at 4 °C overnight, and the thawed milk was transferred to a clean flask located on a clean bench. Milk samples (approximately 1–2 mL each) were collected using a sterile syringe and sent to a clinical laboratory (BML Co., Ltd., Tokyo, Japan) for culture testing. Each batch consisted of breast milk from only one mother.

One hundred-microliter samples from each batch were collected and incubated on blood agar under aerobic, anaerobic, or carbon dioxide-added conditions, or a combination of these conditions (depending on the target bacteria) at 32 ± 10 °C or 35 ± 10 °C for 48 ± 2 h. Bacterial growth was identified by Gram staining, structural morphology, and biochemical tests. Culture methods were established in accordance with HMBANA guidelines. Bacterial counts were expressed as colony-forming units (CFU)/mL. These processes were also conducted in accordance with HMBANA guidelines [11].

### Statistical analyses

The normality and variability of all parameters were evaluated with the Kolmogorov–Smirnov test and F-test, respectively. The total bacterial count and bacterial species count per batch are presented as the median (interquartile range [IQR]); these values were compared between term and preterm HM using the Mann–Whitney U test. For bacterial loads, the number of batches of HM containing specific bacteria among all batches of donated HM is presented as a percentage; this value was compared between the two groups using the Chi-square test or Fisher’s exact test. The total amount of donated HM disposal was analyzed using the Mann–Whitney U test, and the rate of milk sample disposal was analyzed using the Chi-square test.

Donor age, infant birthweight and gestational age, start date of HM donation (calculated using expression and delivery dates of donated milk), and the number of times milk samples were donated per mother are presented as mean (standard deviation) and median (IQR) values; these data were compared between two groups using the Mann–Whitney U test. All statistical analyses were performed using StatMate V (ATMS Co., Ltd., Tokyo, Japan).

## Results

### Characteristics of study participants

The characteristics of all donors are shown in Table 1. Among the 47 registered donors, 31 were term donors, and 16 were preterm donors. The total amount of donated milk batches was 214, including 75 batches from term donors and 139 batches from preterm donors. In some instances, a single donor provided milk multiple times during the study period. There was no significant difference in mean maternal age between the groups (term: 32.3 years vs. preterm: 32.6 years; *p* = 0.75), but there were significant differences in infant gestational ages and birthweights (term: 38.8 weeks vs. preterm: 27.6 weeks; term: 3,084 g vs. preterm: 1,040 g; *p* < 0.001). Additionally, mothers who gave birth to preterm infants tended to begin donating breast milk earlier (term: 18.9 weeks vs. preterm: 9.8 weeks; *p* < 0.001) and more frequently (term: 2.4 times vs. preterm: 8.7 times; *p* < 0.001).

**Table 1:** Relevant details of participants

	**Term**				**Preterm**				***P*-value**
	**n**	**%**	**Mean (SD)**	**Median (IQR)**	**n**	**%**	**Mean (SD)**	**Median (IQR)**	
Number of participants	31				16				
Number of times breast milk was donated by a single donor during the study period	75		2.4 (3.1)	1 (1–2.5)	139		8.7 (8.1)	6.5 (2–10.3)	< 0.001***
Number of disposal milk samples	17	22.7			34	24.5			0.77
Amount of breast milk disposal (ml)			520 (417.3)	455 (120–897.5)			927.5 (297.6)	990 (772.5–1150)	< 0.01**
Age (years)			32.3 (4.9)	32.5 (28–35)			32.6 (4.7)	32.5 (28.8–36.3)	0.75
Gestational age (weeks)			38.8 (1.2)	39 (38–40)			27.6 (3.9)	28.5 (23.8–31)	< 0.001***
Birthweight (g)			3,084 (322.3)	3,099 (2,884–3,290)			1,040 (601.4)	867 (571–1,414.8)	< 0.001***
Chronological age of infant when breast milk was first donated (weeks)			18.9 (6.8)	17 (13–23)			9.8 (5.2)	9 (6–12)	< 0.001***

*P*-values were shown in the table for the Chi-square test and Mann–Whitney U test results

**: < 0.01. ***: < 0.001

### Total bacterial count and bacterial species count

The median total bacterial counts were 3,930 CFU/mL in the term group and 26,700 CFU/mL in the preterm group (Table 2). There was a significant difference in total bacterial count between the two groups (*p* < 0.001). In total, 29 bacterial species were detected in the batches from both groups. The bacterial species count was significantly different in the preterm group (*p* < 0.001). The disposal rate did not significantly differ between preterm HM and term HM (term: 22.7% vs. preterm: 24.5%; *p* = 0.77). However, the median total amount of disposal was greater in the preterm group than in the term group (term: 455 mL vs. preterm: 990 mL; *p* < 0.01) (Table 1).

**Table 2:** Total bacterial count and bacterial species count per batch

	**Term (*n* = 75)**	**Preterm (*n* = 139)**	***P*-value**
Total bacterial count (CFU/mL)	3,930 (435–23,365)	26,700 (4,050–334,650)	< 0.001
Bacterial species count	2 (1–3)	3 (2–3)	< 0.001

Seventy-five batches of human milk (HM) samples were collected from 31 term mothers; 139 batches of HM were collected from 16 preterm mothers. Data represent median (IQR). The total bacterial count and bacterial species count per batch were compared between groups using the Mann–Whitney U test

### Bacterial load

**Fig. 1:**
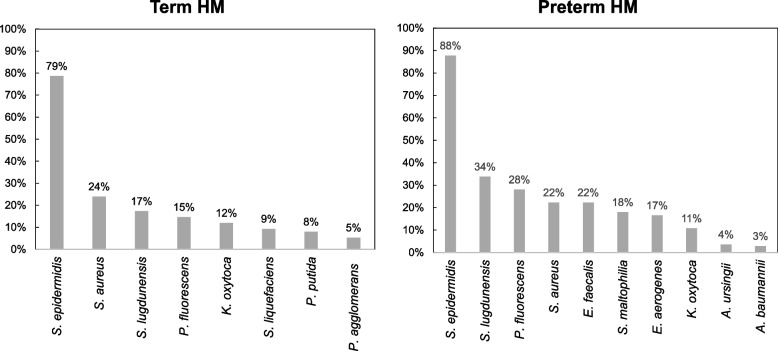
Common bacterial species isolated from term and preterm human milk. Graphs show the proportions of batches of HM containing a specific bacteria that were detected among all batches of donated HM, stratified according to bacterial species. Only the bacterial species with the high proportions are shown. For example, *Staphylococcus epidermidis* was isolated from 59 of 75 batches of term HM (i.e., 79% of batches)

The bacterial loads of HM are shown in Table 3. *Staphylococcus epidermidis* was the most prevalent bacteria (term: 78.7% of batches, preterm: 87.8% of batches), followed by *Staphylococcus lugdunensis* (17.3%, 33.8%), *Staphylococcus aureus* (24.0%, 22.3%), and *Pseudomonas fluorescens* (14.7%, 28.1%), all of which were detected at high frequencies in both groups (Fig. 1).

**Table 3:** Type of bacteria and bacterial load in donated human milk samples

**Bacteria**	**Term (*n* = 75)**		**Preterm (*n* = 139)**		***P*-value**
	**n**	**Prevalence (%)**	**n**	**Prevalence (%)**	
*Staphylococcus epidermidis*	59	78.7%	122	87.8%	0.079
*Staphylococcus lugdunensis*	13	17.3%	47	33.8%	0.01*
*Staphylococcus aureus*	18	24.0%	31	22.3%	0.78
*Pseudomonas fluorescens*	11	14.7%	39	28.1%	0.027*
*Acinetobacter baumannii*	1	1.3%	4	2.9%	0.66
*Enterococcus faecalis*	-	-	31	22.3%	< 0.001***
*Enterobacter aerogenes*	-	-	23	16.5%	< 0.001***
*Stenotrophomonas maltophilia*	3	4.0%	25	18.0%	0.003**
*Pseudomonas* sp.	3	4.0%	3	2.2%	0.43
*Pseudomonas putida*	6	8.0%	1	0.7%	0.008**
*Pseudomonas aeruginosa*	2	2.7%	-	-	0.12
*Serratia liquefaciens*	7	9.3%	-	-	< 0.001***
*Serratia* sp.	-	-	2	1.4%	0.77
*Pantoea agglomerans*	4	5.3%	-	-	0.014*
*Raoultella ornithinolytica*	1	1.3%	2	1.4%	1
*Acinetobacter johnsonii*	3	4.0%	1	0.7%	0.13
*Acinetobacter* sp.	1	1.3%	1	0.7%	1
*Acinetobacter ursingii*	3	4.0%	5	3.6%	1
*Streptococcus agalactiae*	-	-	2	1.4%	0.77
*Escherichia coli*	1	1.3%	-	-	0.35
*α-streptococcus*	2	2.7%	1	0.7%	0.28
*Klebsiella oxytoca*	9	12.0%	15	10.8%	0.79
*Klebsiella pneumoniae*	-	-	1	0.7%	1
*Enterobacter* sp.	3	4.0%	1	0.7%	0.13
*Bacillus cereus*	3	4.0%	-	-	0.042*
*Bacillus subtilis*	1	1.3%	-	-	0.35
*Rhizobium radiobacter*	-	-	1	0.7%	1
*Staphylococcus warneri*	1	1.3%	1	0.7%	1
*Staphylococcus haemolyticus*	1	1.3%	-	-	0.35

Bacterial load refers to the number of batches of milk containing certain bacteria, that were detected out of the total batches of donated human milk (HM)

In some batches, two or more bacteria were detected

*P*-values were shown in the table for the Chi-squared test or Fisher’s exact test results of the bacterial load for HM batches from term and preterm mothers

*: < 0.05, **: < 0.01. ***: < 0.001

Four bacterial species were more prevalent in term HM: *Pseudomonas putida* (*p* = 0.008), *Serratia liquefaciens* (*p* < 0.001), *Pantoea agglomerans* (*p* = 0.014), and *Bacillus cereus* (*p* = 0.042). In contrast, five species were more prevalent in preterm HM: *Pseudomonas fluorescens* (*p* = 0.027)*, Enterococcus faecalis* and *Enterobacter aerogenes* (*p* < 0.001)*, Staphylococcus lugdunensis* (*p* = 0.01)*,* and *Stenotrophomonas maltophilia* (*p* = 0.003) (Table 3).

## Discussion

Our bacterial culture studies of term and preterm HM revealed three major findings. First, the bacterial species detected at high frequencies were common to both groups. *Staphylococcus epidermidis*, a commensal skin bacterium, was the most frequent potential bacterial pathogen in both groups; it was detected in approximately 80% of batches. Coagulase-negative staphylococci (CoNS), *Staphylococcus aureus*, and *Pseudomonas fluorescens* were also found at high rates. These results are consistent with findings in previous studies [14–17], confirming that these common commensal bacteria are likely to be isolated from HM, regardless of the donor’s living environment.

Second, the total bacterial count was significantly higher in preterm HM than in term HM, and the bacterial species count was greater in preterm HM.

Third, there were differences in bacterial profiles between term and preterm HM. The characteristics of four bacterial species in term HM are as follows. *Serratia liquefaciens* and *Pseudomonas putida* are often isolated from water and soil environments [18, 19]; notably, *Pseudomonas putida* is rarely isolated from clinical specimens [19]. *Pantoea agglomerans* is a Gram-negative bacterium commonly present in fecal material and soil, but it is an uncommon cause of infection in children [20]. The spore-forming bacterium *Bacillus cereus* was detected in three batches. *Enterococcus faecalis* and *Enterobacter aerogenes* were more frequently isolated from preterm HM. These bacteria are classified as enterococci that reside in the human gastrointestinal tract, and they are frequently reported to cause nosocomial infections [21, 22]. There have been reports of enterococcal outbreaks in NICUs related to tap water contamination [23]. *Pseudomonas fluorescens* is widely found in water supplies; it can also be isolated from medical devices [24]. *Staphylococcus lugdunensis*, a CoNS species, is sometimes clinically managed in a manner identical to *Staphylococcus aureus* [25]. It is a commensal skin bacterium [26] and is reportedly a common cause of community-acquired and nosocomial infections [25, 27]. *Stenotrophomonas maltophilia* is commonly isolated from water, soil, and fecal material; it is often detected in hospitals, particularly in the water supply [23, 28].

According to a report by Urrea et al. [29, 30], *Enterococcus* species, *Staphylococcus aureus,* and CoNS such as *Staphylococcus epidermidis* are the most common Gram-positive bacteria responsible for nosocomial infections in NICU; *Escherichia coli*, *Enterobacter* species, *Pseudomonas* species, and *Klebsiella* species are the most common Gram-negative bacteria responsible for nosocomial infections in NICU. Of the five species present at significantly different rates in the preterm HM, four species were associated with the bacterial species identified in the previous report. This result suggests that preterm HM tends to contain bacteria that can cause nosocomial infections in NICUs.

Based on the results of this study, there were several insights. First, preterm donors visit the NICU to meet their infants; therefore, they have a high risk of exposure to bacteria that are prevalent in the NICU environment. Accordingly, there is a potential risk that such bacteria will be present in HM from preterm donors, expressed either in the NICU or at home. A report by Beghetti et al. also suggested that hospital exposure influences the bacterial profile in preterm HM [31]. It is likely that all donors expressed at home because the study period coincided with the coronavirus disease 2019 pandemic, and many NICUs had restricted visiting hours. Although detailed information regarding the expression environment (location and methods) was not collected in this study, this factor also may have significantly influenced HM microbiota.

Second, several preterm HM samples included HM that had been expressed before donor registration. As a basic premise, HMBs always provide hygiene instructions (e.g., pre-breast expression wiping and disinfection of the breast pump) at donor registration, regardless of whether the donor gave birth prematurely. Term mothers may voluntarily register as a donor if they delivered a term baby, are currently breastfeeding, and have excess breast milk supply. After registration, they donate milk to the HMB. However, preterm mothers may provide milk to HMBs; this HM is held in reserve for infants admitted to NICUs. Importantly, the milk is expressed by mothers have not received instruction regarding hygiene procedures at HMBs. Thus, there is a higher risk that preterm HM contains a high bacterial load. In Japan, preterm mothers can ask NICU staff to store their breast milk in fridges in the NICU until the maximum volume (10 L) is reached; the milk is then shipped to the HMB. Regarding the effect of freezing on the numbers of bacteria in breast milk, storage at 4 °C (i.e., refrigeration) or freezing are recommended methods for breast milk preservation from a bacteriological perspective; notably, freezing decreases rather than increases the number of bacteria [32]. Furthermore, there is reportedly no difference in numbers of bacteria between frozen breast milk and typical milk deposits received at HMBs [33]; we suspect that the numbers of bacteria may also be similar between typical milk deposits and frozen milk that has been stored in the NICU. Therefore, hygienic expression procedures may be an important influencing factor. The JHMBA has a uniform storage period for donor HM (pasteurized within 3 months from the expression date), regardless of whether the donor is a term mother or preterm mother; accordingly, breast milk stored by preterm mothers prior to donor registration is also within the regulated storage period.

Third, the unique circumstances of preterm donors should also be considered. Several factors contribute to a stressful expression environment for preterm donors, including physical separation from their infants, the provision of a structured feeding schedule, the lack of privacy (when expressing in a hospital), the exhaustion and anxiety associated with an infant’s hospitalization, and long expression periods. These factors can also affect HM production [34–37]. Considering the situation, it is understandably more difficult to consider hygiene precautions when expressing in a hospital than when expressing at home, and the resulting HM may contain more bacteria.

Furthermore, there is a possibility that pre-pregnancy body-mass index and delivery mode influence temporal changes in the microbiota of HM from preterm mothers [38]. Although these factors were not considered in the present study, they may have contributed to the characteristics of preterm HM observed in this study.

The results of this pilot study indicate that there are differences in the numbers and types of bacteria cultured from HM between mothers of term infants who are at home and mothers of preterm infants who are hospitalized in NICUs. Maternal lifestyle and environment may also have influenced the results. Based on these findings, there is a need to explore methods to reduce the disposal of donated HM, particularly from preterm donors. Additionally, these results indicate that hygiene education is more important for preterm donors. However, their physical and psychological circumstances should be considered. HMBs should provide less burdensome and more hygienic expression instructions for these mothers. It may also be necessary to communicate more frequently with preterm donors and to supplement the instructions with observations of their milk expression and storage methods at home. Additionally, it may be necessary to survey the NICU situation at each institution and discuss hygiene instructions for preterm donors with NICU staff.

Although the results of this study did not show a significant difference in the pass/fail score according to bacterial culture test criteria established by the HMB (Table 1), in the future, better hygiene instructions will reduce the risk of infection transmission via breast milk; they will also help to reduce the disposal of valuable donated HM, an important issue for HMBs. In addition to hygiene instructions, there may be a need to reconsider donor HM eligibility criteria, particularly with respect to preterm HM.

## Limitations

The HMB system in Japan was initiated in 2017, and the number of recipients was approximately 200 in 2021. Thus, the HMB system in Japan is in its infancy, compared with other developed countries. Accordingly, the number of donors was small during this study, such that only 16 preterm donors were recruited (approximately only about half of the total number of term donors). Despite this limitation, we analyzed a total of 214 HM samples to more fully determine how bacterial counts and profiles differed between term and preterm mothers. Although this was a small pilot study, it was the first study to focus on Japanese donors; the results regarding bacterial species in term and preterm mothers are consistent with published reports. Preterm HM is known to be better matched nutritionally for preterm infants [1–3]; therefore, it is important to reduce the disposal of donated HM from preterm donors. The small number of donors does not reduce the importance of the results obtained from the first HMB in Japan.

The details of expression methods were not investigated; thus, the impacts of these factors on the culture results are unknown. Various other factors, such as living arrangements, sibling status, maternal body-mass index and delivery mode, and temporal effects, may also affect the bacterial profile of HM and will be investigated in the future. Although HM bacteria were measured using the culture method in this study, future studies will involve additional participants and more extensive microbiological studies with metagenomic analyses.

## Conclusions

This study revealed that preterm HM has a higher total bacterial count than term HM and suggested that preterm HM tends to have more nosocomial-infection-causing bacteria in the NICU. Taken together, these findings suggest the need for more focused hygiene education for preterm donors.

## Data Availability

The datasets used and / or analyzed during the current study are available from the corresponding author on reasonable request.

## Abbreviations

HM: Human milk
NICUs: Neonatal Intensive Care Units
CFU: Colony-forming units
HMB: Human milk bank
JHMBA: Japan Human Milk Bank Association
HMBANA: Human Milk Banking Association of North America
CoNS: Coagulase-negative Staphylococci
